# Analysis of influencing factors of pulmonary infection during chemotherapy in patients with multiple myeloma and construction of a nomogram prediction model

**DOI:** 10.1016/j.clinsp.2025.100822

**Published:** 2025-11-12

**Authors:** Tianxia Deng, Hui Zhong, Xiaohong Xie, Jinglong Lv

**Affiliations:** Department of Hematology, Chongqing University Three Gorges Hospital, Chongqing 404100, China

**Keywords:** Multiple myeloma, Chemotherapy, Pulmonary infection, Influencing factors, Nomogram

## Abstract

**Objective:**

To identify risk factors for pulmonary infection during chemotherapy in Multiple Myeloma (MM) patients and construct a nomogram prediction model.

**Methods:**

A total of 171 MM patients admitted from September 2022 to September 2024 were retrospectively analyzed and divided into modeling and validation groups (7:3) using the random number table method. The modeling group was further classified by the occurrence of pulmonary infection. Multivariate logistic regression was applied to identify risk factors, and R software was used to develop the nomogram.

**Results:**

Among 171 patients, 89 (52.05 %) developed pulmonary infections. In the modeling group (*n* = 120), the incidence was 55.00 % (66 cases). Multivariate analysis showed that age, International Staging System (ISS) stage, Eastern Cooperative Oncology Group (ECOG) score, anemia, granulocytopenia, and albumin level were significant risk factors (*p* < 0.05). The nomogram demonstrated good discrimination, with an AUC of 0.883 in the modeling group and 0.880 in the validation group. Calibration curves indicated strong agreement between predicted and observed outcomes, with Hosmer-Lemeshow (H-L) test results of χ^2^ = 6.912, *p* = 0.697, and χ^2^ = 6.756, *p* = 0.642, respectively. Decision Curve Analysis (DCA) confirmed high clinical utility when the threshold probability ranged from 0.08 to 0.92.

**Conclusion:**

Age, ISS stage, ECOG score, anemia, granulocytopenia, and albumin level significantly influence pulmonary infection risk during MM chemotherapy. The nomogram provides an effective tool for individualized risk prediction.

## Highlights

• Pulmonary infection occurred in 52.05 % of MM patients during chemotherapy.
• Six key risk factors were identified: age, ISS stage, ECOG score, anemia, neutropenia, and albumin.
• A nomogram model was developed with high accuracy (AUC > 0.88).
• Calibration and decision curve analysis confirmed strong predictive performance.
• The nomogram enables individualized risk prediction for MM chemotherapy patients.

## Introduction

Multiple Myeloma (MM) is a malignant proliferative tumor originating from plasma cells, predominantly occurring in middle-aged and elderly individuals. It has a low incidence rate but a high mortality rate, severely impacting patients' lives.^1^ Chemotherapy is an important clinical treatment for MM, effectively improving patient prognosis and extending survival time. However, while chemotherapy drugs kill tumor cells, they can also damage normal cells, leading to reduced immune function and increased susceptibility to various infections, which negatively affect prognosis.^2^^,^^3^ Pulmonary infection is one of the most common complications in MM patients, which can exacerbate the disease condition.^4^ Therefore, preventing and treating pulmonary infections in MM patients, identifying the influencing factors during chemotherapy, and timely interventions are crucial for improving patient prognosis. Nomograms can analyze the identified risk factors to predict the risk value of an event, quantify the risk prediction, and enable clinical physicians to formulate corresponding intervention measures based on the risk factors, thereby effectively reducing the risk of gastrointestinal bleeding.^5^ Based on this, there are currently few reports on nomogram studies of pulmonary infections occurring during chemotherapy in MM patients. This study aims to explore the influencing factors of pulmonary infections during chemotherapy in MM patients and construct a nomogram prediction model for this condition.

## Materials and methods

### General data

**Fig. 1:**
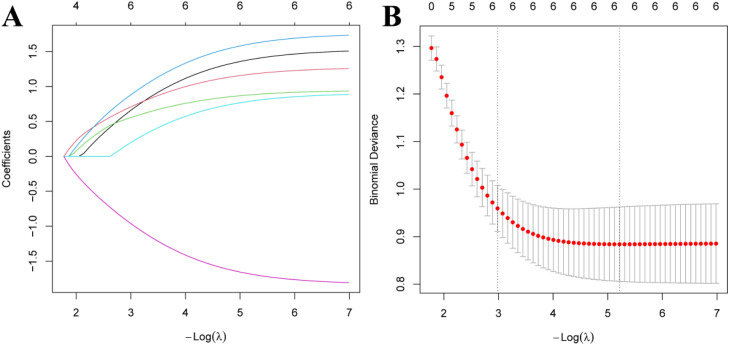
Factors affecting pulmonary infection during MM chemotherapy (A) Lasso regression coefficient relationship diagram; (B) Lasso regression 10-fold cross-validation results.

A total of 171 MM patients admitted to the hospital between September 2022 and September 2024 were retrospectively included. Patients were randomly assigned to a modeling group (120 cases) and a validation group (51 cases) in a 7:3 ratio (randomization was performed using a random number table, and allocation concealment was ensured with opaque, sealed envelopes). Patients in the modeling group were further divided into a pulmonary infection group and a non-pulmonary infection group based on whether they developed pulmonary infections during chemotherapy. The case collection flowchart is shown in Fig. 1. Inclusion criteria: 1) Meeting MM diagnostic criteri;a^6^ 2) Meeting pulmonary infection diagnostic criteria; 3) First occurrence of MM; 4) Complete clinical data available. Exclusion criteria: 1) Major organ failure; 2) Presence of malignant tumors; 3) Pre-existing pulmonary disease; 4) Hematologic diseases; 5) History of infection before chemotherapy; 6) Mental illness or cognitive impairment. This study was approved by the hospital’s ethics committee.

### Pulmonary infection diagnosis^7^

Pulmonary infection can be diagnosed if any of the first four criteria were present in combination with the fifth: 1) WBC > 10 × 10⁹/L or < 4 × 10⁹/L; 2) Body temperature ≥ 38 °C, accompanied by fever; 3) Worsened pre-existing respiratory symptoms or new symptoms such as cough; 4) Wet rales or lung consolidation detected on auscultation; 5) Imaging showing lung lesions, possibly with pleural effusion.

### Clinical data

Clinical data were collected from the electronic medical records, including age, gender, Body Mass Index (BMI), hypertension, diabetes, renal insufficiency, smoking history (continuous or cumulative smoking for more than six months), alcohol consumption history (average consumption of 50 g per session for ≥1-year), International Staging System (ISS) stage,^8^ Eastern Cooperative Oncology Group (ECOG) performance status score,^9^ anemia, complications, albumin levels, central venous catheterization, Neutrophil-to-Lymphocyte Ratio (NLR), Monocyte-to-Lymphocyte Ratio (MLR), Controlling Nutritional Status (CONUT) score,^10^ and Prognostic Nutritional Index (PNI) score.^11^

### Data collection methods

All personnel involved in the study underwent standardized training to ensure a clear understanding of the scale items. Data collection was performed using the hospital's electronic medical record system, and a pathological data investigation form was designed. Data collectors were proficient in using the electronic medical record system and had at least five years of professional experience. Each item was verified to ensure the validity and authenticity of the data.

### Statistical analysis

Data analysis was performed using SPSS 25.0. Categorical data were analyzed using the χ^2^ test and expressed as cases (%). Measurement data conforming to normal distribution were analyzed using the *t*-test and expressed as ($\bar{x}\pm S$). Lasso regression was applied to select relevant variables. Risk factors for pulmonary infections during chemotherapy in MM patients were analyzed using multivariate logistic regression. *R* software (version 4.5.1, rms package) was used to construct the nomogram model. The ROC curve was used to evaluate the discrimination of the nomogram model, and the Decision Curve Analysis (DCA) curve was used to assess its clinical utility. A p-value < 0.05 was considered statistically significant.

## Results

### Comparison of clinical data between the modeling group and the validation group

There were no significant differences in clinical data between the modeling group and the validation group (*p* > 0.05). See Table 1.

**Table 1:** Comparison of clinical data between the modeling group and the validation group.

Factor	Modeling group (*n* = 120)	Validation group (*n* = 51)	*χ*^2^	p
Age (years)			0.032	0.859
< 60	50 (41.67)	22 (43.14)		
≥ 60	70 (58.33)	29 (56.86)		
Genders			0.212	0.645
Man	66 (55.00)	30 (58.82)		
Woman	54 (45.00)	21 (41.18)		
BMI (kg/m^2^)			0.385	0.535
< 24	74 (61.67)	34 (66.67)		
≥ 24	46 (38.33)	17 (33.33)		
Hypertension			0.214	0.644
Yes	20 (16.67)	10 (19.61)		
No	100 (83.33)	41 (80.39)		
Diabetes			0.418	0.518
Yes	23 (19.17)	12 (23.53)		
No	97 (80.83)	39 (76.47)		
Renal insufficiency			0.091	0.762
Yes	28 (23.33)	13 (25.49)		
No	92 (77.67)	38 (74.51)		
Smoking history			0.176	0.675
Yes	43 (35.83)	20 (39.22)		
No	77 (64.17)	31 (60.78)		
Drinking history			0.160	0.689
Yes	34 (28.33)	16 (31.37)		
No	86 (71.67)	35 (68.63)		
ISS staging			0.045	0.833
I∼II stage	45 (37.50)	20 (39.22)		
III stage	75 (62.50)	31 (60.78)		
ECOG score (points)			0.045	0.832
< 2	52 (43.33)	23 (45.10)		
≥ 2	68 (56.67)	28 (54.90)		
Anemic			0.237	0.626
Yes	61 (50.83)	28 (54.90)		
No	59 (49.17)	23 (45.10)		
Complications			0.105	0.746
Yes	55 (45.83)	22 (43.14)		
No	65 (54.17)	29 (56.86)		
Albumin (g/L)			0.159	0.690
< 35	51 (42.50)	20 (39.22)		
≥ 35	69 (57.50)	31 (60.78)		
Deep vein cannulation			0.555	0.456
Yes	18 (15.00)	10 (19.61)		
No	102 (85.00)	41 (80.39)		
NLR			0.150	0.698
< 2	62 (51.67)	28 (54.90)		
≥ 2	58 (48.33)	23 (45.10)		
MLR			0.146	0.702
< 0.3	65 (54.17)	26 (50.98)		
≥ 0.3	55 (45.83)	25 (49.02)		
CONUT score (points)			0.031	0.860
< 2	43 (35.83)	19 (37.25)		
≥ 2	77 (64.17)	32 (62.75)		
PIN score (points)			0.314	0.575
< 45	55 (45.83)	21 (41.18)		
≥ 45	65 (54.17)	30 (58.82)		

### Comparison of clinical data between the pulmonary infection group and the non-pulmonary infection group

Out of 171 patients, 89 experienced pulmonary infections, with an incidence rate of 52.05 %. Among the 120 patients in the modeling group, 66 developed pulmonary infections, with an incidence rate of 55.00 %. Significant differences were observed between the two groups in terms of age, ISS stage, ECOG score, anemia, neutropenia, and albumin levels (*p* < 0.05). No significant differences were found in other clinical data (*p* > 0.05). See Table 2.

**Table 2:** Comparison of clinical data between the lung infection group and non-lung infection group.

Factor	Lung Infection Group (*n* = 66)	Non lung infection group (*n* = 54)	*χ*^2^	p
Age (years)			12.502	<0.001
< 60	18 (27.27)	32 (59.26)		
≥ 60	48 (72.73)	22 (40.74)		
Genders			0.067	0.796
Man	37 (56.06)	29 (53.70)		
Woman	29 (43.94)	25 (46.30)		
BMI (kg/m^2^)			0.013	0.910
< 24	41 (62.12)	33 (61.11)		
≥ 24	25 (37.88)	21 (38.89)		
Hypertension			0.242	0.622
Yes	12 (18.18)	8 (14.81)		
No	54 (81.82)	46 (85.19)		
Diabetes			0.027	0.877
Yes	13 (19.70)	10 (18.52)		
No	53 (80.30)	44 (81.48)		
Renal insufficiency			0.068	0.795
Yes	16 (24.24)	12 (22.22)		
No	50 (75.76)	42 (77.78)		
Smoking history			0.267	0.605
Yes	25 (37.88)	18 (33.33)		
No	41 (62.12)	36 (66.67)		
Drinking history			0.280	0.597
Yes	20 (30.30)	14 (25.93)		
No	46 (69.70)	40 (74.07)		
ISS staging			10.999	0.001
I∼II stage	16 (24.24)	29 (53.70)		
III stage	50 (75.76)	25 (46.30)		
ECOG score (points)			10.141	0.001
< 2	20 (30.30)	32 (59.26)		
≥ 2	46 (69.70)	22 (40.74)		
Anemic			12.031	0.001
Yes	43 (65.15)	18 (33.33)		
No	23 (34.85)	36 (66.67)		
Complications			0.076	0.782
Yes	31 (46.97)	24 (44.44)		
No	35 (53.03)	30 (55.56)		
Albumin (g/L)			13.916	<0.001
< 35	18 (27.27)	33 (61.11)		
≥ 35	48 (72.73)	21 (38.89)		
Deep vein cannulation			0.003	0.959
Yes	10 (15.15)	8 (14.81)		
No	56 (84.85)	46 (85.19)		
NLR			0.487	0.485
< 2	36 (54.55)	26 (48.15)		
≥ 2	30 (45.45)	28 (51.85)		
MLR			0.212	0.645
< 0.3	37 (56.06)	28 (51.85)		
≥ 0.3	29 (43.94)	26 (48.15)		
CONUT score (points)			1.951	0.162
< 2	20 (30.30)	23 (42.59)		
≥ 2	46 (69.70)	31 (57.41)		
PIN score (points)			0.212	0.645
< 45	29 (43.94)	26 (48.15)		
≥ 45	37 (56.06)	28 (51.85)		

### Analysis of influencing factors for pulmonary infections during chemotherapy in MM patients

The modeling group of MM patients was used, with pulmonary infection occurrence during chemotherapy in MM patients was set as the dependent variable (yes = 1, no = 0), while age, ISS stage, ECOG score, anemia, neutropenia, and albumin levels were set as independent variables. The variable assignments are shown in Table 3. Lasso analysis was performed using *R* software, with ten-fold cross-validation applied to determine the optimal λ value. The results showed that when the penalty coefficient λ = 0.05184687, the model achieved good performance with a minimal number of influencing factors (see Fig. 1). Collinearity among the six influencing factors was assessed, and all Variance Inflation Factors (VIFs) were < 10, indicating no multicollinearity or interaction among the factors. Multivariate logistic regression analysis indicated that age, ISS stage, ECOG score, anemia, neutropenia, and albumin levels were risk factors for pulmonary infections during chemotherapy in MM patients (*p* < 0.05). See Table 4, Table 5.

**Table 3:** Assignment methods of argument variables.

Variable	Assignment method
Age	< 60-years old = 0, ≥ 60-years old = 1
ISS staging	I∼II stage = 0, III stage = 1
ECOG score	< 2 points = 0, ≥ 2 points = 1
Anemic	yes = 1, no = 0
Granulocyte deficiency	yes = 1, no = 0
Albumin	≥ 35 g/*L* = 1, < 35 g/*L* = 0

**Table 4:** Univariate analysis of pulmonary infections during chemotherapy in patients with MM.

Variable	β value	SE variable	Wald χ^2^ variable	p-variable	OR variable	95 % CI
Age	1.415	0.342	17.139	<0.001	4.120	2.108∼8.054
ISS staging	1.103	0.413	7.136	0.008	3.014	1.341∼6.772
ECOG score	1.083	0.342	10.031	0.002	2.954	1.511∼5.775
Anemic	1.139	0.402	8.029	0.005	3.124	1.421∼6.869
Granulocyte deficiency	1.271	0.436	8.496	0.004	3.564	1.516∼8.377
Albumin	0.977	0.416	5.518	0.019	2.657	1.176∼6.005
Constant	−4.423	0.703	39.582	<0.001	0.012	‒

**Table 5:** Multivariate analysis of lung infection during chemotherapy in MM patients.

Variable	β value	SE variable	Wald χ^2^ variable	p-variable	OR variable	95 % CI
Age	1.678	0.516	10.588	0.001	5.357	1.949∼14.723
ISS staging	1.224	0.531	5.321	0.021	3.400	1.202∼9.617
ECOG score	1.102	0.304	13.139	<0.001	3.010	1.659∼5.462
Anemic	1.227	0.515	5.675	0.017	3.413	1.243∼9.369
Granulocyte deficiency	1.322	0.530	6.225	0.013	3.752	1.328∼10.601
Albumin	1.087	0.523	4.317	0.038	2.967	1.064∼8.275
Constant	−3.619	0.714	25.719	<0.001	0.027	‒

### Construction of a nomogram model for predicting pulmonary infections during chemotherapy in MM patients

**Fig. 2:**
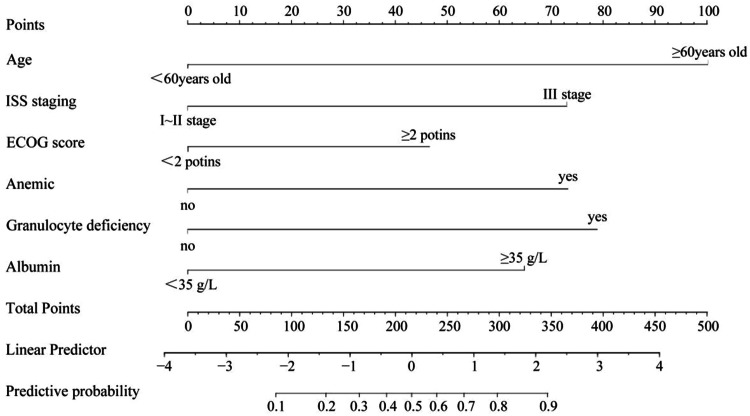
Development of a nomogram model of lung infections occurring during chemotherapy in MM patients.

A nomogram model was constructed based on the identified risk factors, with the predicted probability calculated as *P* = eˣ / (1 + eˣ), where *x* = –3.619 + 1.678 × age + 1.224 × ISS stage + 1.102 × ECOG score + 1.227 × anemia + 1.322 × neutropenia + 1.087 × albumin level. In the model, the factors influencing the score in descending order were age, neutropenia, ISS stage, anemia, albumin levels, and ECOG score. For example, a patient aged < 60-years (0-points), with ISS stage III (73.5-points), ECOG score < 2 (0-points), anemia (72.0-points), neutropenia (79.5-points), and albumin levels ≥ 35 g/L would have a total score of 290.5 points. A vertical line drawn from this total score predicts a probability of 79 %. See Fig. 2.

### Nomogram model for the modeling group

**Fig. 3:**
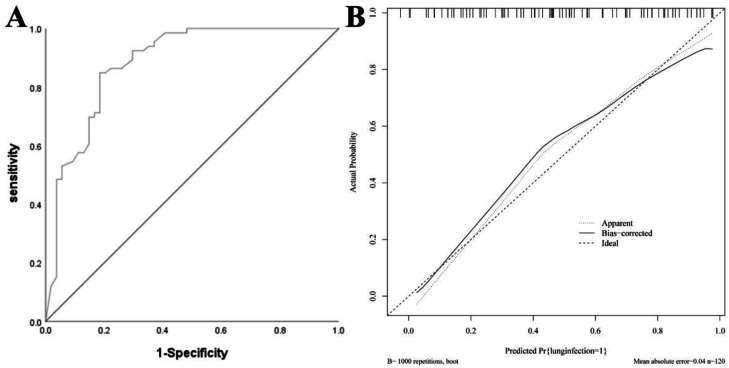
Nomogram Model for the Modeling Group. (A) ROC curve of modeling group; (B) Calibration curve of modeling group.

The AUC of the modeling group was 0.883 (95 % CI: 0.820–0.946), and the calibration curve demonstrated strong agreement between predicted and observed outcomes. The H-L test yielded χ^2^ = 6.912, *p* = 0.697, indicating good calibration. See Fig. 3.

### Nomogram model for the validation group

**Fig. 4:**
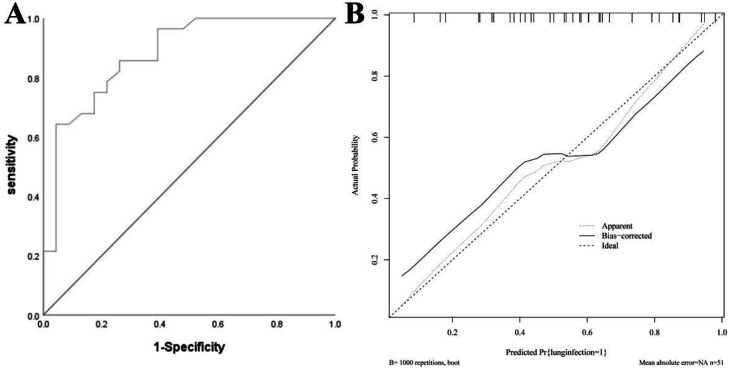
Nomogram Model for the Validation Group. (A) ROC curve of the validation group; (B) Calibration curve of the validation group.

The AUC of the validation group was 0.880 (95 % CI: 0.785–0.974), and the calibration curve demonstrated strong agreement between predicted and observed outcomes. The H-L test yielded χ^2^ = 6.756, *p* = 0.642, indicating good calibration. See Fig. 4.

### DCA curve of the nomogram model

**Fig. 5:**
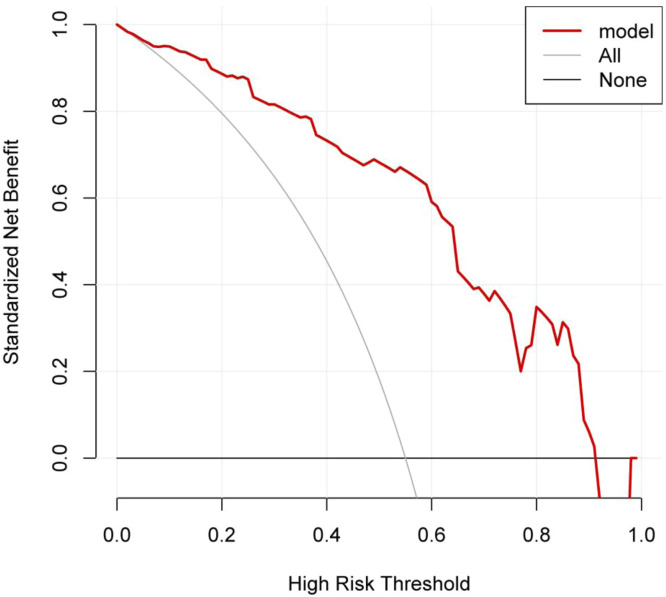
DCA curve for the nomogram. Note: The X-axis represents the high-risk threshold, the Y-axis represents the net benefit, the lower black line represents the patient's net benefit from developing a lung infection, and the oblique gray line represents the patient's net benefit from developing a lung infection.

The DCA curve showed that the clinical utility of predicting pulmonary infections during chemotherapy in MM patients was high when the probability was between 0.08 and 0.92. See Fig. 5.

## Discussion

MM, as a malignant plasma cell clonal disease, leads to immune dysfunction in patients due to the damage caused by the immunoglobulins and fragments secreted by abnormally proliferating plasma cells in the bone marrow.^12^ Pulmonary infection is a frequent complication in MM patients undergoing chemotherapy, as impaired immune function during treatment creates favorable conditions for pathogen invasion.^13^ Studies have found that pulmonary infections in MM patients after chemotherapy may be caused by the stimulation of bone marrow stroma, inducing the expression of Interleukin-6 (IL-6) and vascular endothelial growth factor, which obstruct the generation of polyclonal antibody pathways, lower the CD4+/CD8+ ratio, and reduce the bioactivity of dendritic cells, thereby disrupting the immune microenvironment and increasing the risk of pulmonary infection.^14^^,^^15^ The results of this study showed that among 171 patients, 89 developed pulmonary infections, yielding an incidence rate of 52.05 %. In the modeling group of 120 patients, 66 cases were recorded, corresponding to an incidence rate of 55.00 %, which reflects a relatively high frequency. Therefore, establishing a risk prediction model for pulmonary infections during chemotherapy in MM patients is of considerable clinical significance. This study identified six influencing factors through Lasso regression analysis. The reasons are analyzed as follows: 1) Age: The older the MM patient, the more severe the dysfunction of their organ systems becomes. Additionally, reduced immune function in the later stages of treatment, prolonged hospital stays, and increased opportunities for cross-infection with pathogens collectively increase the risk of infection, making older patients more susceptible to pulmonary infections.^16^ 2) ISS Stage: Patients in ISS stage III experience an increasing tumor burden in the bone marrow, leading to immune imbalance and uncontrolled infections, thereby raising the risk of infection.^17^ 3) ECOG Score: A higher ECOG score reflects a more severe condition in MM patients, accompanied by more pronounced tumor-induced immune deficiencies. Alterations in the immune microenvironment contribute to a gradual rise in pulmonary infection rates. Furthermore, chemotherapy further compromises immune function, thereby elevating the risk of pulmonary infections.^18^ 4) Anemia: Studies have revealed that red blood cells, apart from facilitating gas exchange, are involved in immune processes. Their surface contains certain innate immune molecules that can recognize, adhere to, and clear circulating immune complexes. Therefore, a reduction in red blood cell count can lead to impaired immune function, suggesting that anemia may be a risk factor for pulmonary infections.^19^ 5) Neutropenia: Neutrophils play a vital role in the nonspecific immunity of the bloodstream. During pulmonary infections, activated neutrophils migrate into the lungs via the pulmonary capillary circulation, aggregating in large numbers to phagocytize and degrade bacteria and tissue debris. They release reactive oxygen species and antimicrobial peptides through respiratory burst, killing pathogens and enhancing immunity. A lack of neutrophils, however, may increase the risk of pulmonary infections. Therefore, the timely administration of granulocyte-stimulating factors in MM patients can help reduce pulmonary infection risks.^20^^,^^21^ 6) Albumin Levels: Reduced albumin levels may be related to IL-6 suppression of hepatocyte albumin synthesis, as IL-6 is negatively correlated with albumin levels and closely associated with MM. During infections, elevated IL-6 levels in MM patients further lower albumin levels, and prolonged hospital stays increase the risk of pulmonary infections. Reduced albumin levels impair immune function and heighten the risk of pulmonary infections.^22^^,^^23^

The nomogram model developed in this study showed AUCs of 0.883 and 0.880 for the modeling and validation groups, respectively, indicating high discriminatory power. The calibration curve slopes were close to 1, demonstrating good prediction consistency. The DCA curve indicated high clinical utility for predicting pulmonary infections during chemotherapy in MM patients when the probability was between 0.08 and 0.92. Therefore, timely attention to the aforementioned factors may help clinicians intervene early based on the identified risk factors. For patients predicted to have a high risk of pulmonary infection during chemotherapy, individualized treatment strategies can be actively developed. The nomogram can assist clinicians in decision-making by quantifying risk through these factors. Presented as a visualized model, it helps clinicians make more rational choices in complex clinical scenarios, thereby effectively improving patient prognosis and enhancing quality of life.

In conclusion, age, ISS stage, ECOG score, anemia, neutropenia, and albumin levels are influencing factors for pulmonary infections during chemotherapy in MM patients. The constructed nomogram model effectively predicts the risk of pulmonary infections. However, this study has several limitations, including being a single-center study with potential selection bias, pulmonary infections not microbiologically confirmed, and no strategy described for handling missing data. The sample size was relatively small, and external validation was not performed separately. Future studies will expand the sample size and scope for further validation.

## Research involving human participants

The study was approved by the Ethics Review Board of Chongqing University Three Gorges Hospital (n° 2025,016) and conducted in accordance with the 1964 Helsinki Declaration and its later amendments. Written informed consent was obtained from all participants.

## Declaration of competing interest

The authors declare no conflicts of interest.

## References

[1] Chicca I.J., Heaney J.L.J., Iqbal G., Dunn J.A., Bowcock S., Pratt G. (2020). Anti-bacterial antibodies in multiple myeloma patients at disease presentation, in response to therapy and in remission: implications for patient management. Blood Cancer J.

[2] Cho Y.-R., Yoo Y.-S. (2020). Factors influencing supportive care needs of multiple myeloma patients treated with chemotherapy. Support Care Cancer.

[3] Rajkumar S.V. (2022). Multiple myeloma: 2022 update on diagnosis, risk stratification, and management. Am J Hematol.

[4] Pawlyn C., Davies F.E. (2019). Toward personalized treatment in multiple myeloma based on molecular characteristics. Blood.

[5] Zhang Y., Zhang Z., Wei L., Wei S. (2022). Construction and validation of nomograms combined with novel machine learning algorithms to predict early death of patients with metastatic colorectal cancer. Front Public Health.

[6] Cowan A.J., Green D.J., Kwok M., Lee S., Coffey D.G., Holmberg L.A. (2022). Diagnosis and management of multiple myeloma: a review. JAMA.

[7] Kalil A.C., Metersky M.L., Klompas M., Muscedere J., Sweeney D.A., Palmer L.B. (2016). Executive summary: management of adults with hospital-acquired and ventilator-associated pneumonia: 2016 Clinical Practice Guidelines by the Infectious Diseases Society of America and the American Thoracic Society. Clin Infect Dis.

[8] Greipp P.R., San Miguel J., Durie B.G.M., Crowley J.J., Barlogie B., Bladé J. (2005). International staging system for multiple myeloma. J Clin Oncol.

[9] Facon T., Dimopoulos M.A., Meuleman N., Belch A., Mohty M., Chen W.-M. (2020). A simplified frailty scale predicts outcomes in transplant-ineligible patients with newly diagnosed multiple myeloma treated in the FIRST (MM-020) trial. Leukemia.

[10] Ignacio de Ulíbarri J., González-Madroño A., De Villar N.G.P., González P., González B., Mancha A. (2005). Conut: a tool for controlling nutritional status. First validation in a hospital population. Nutr Hosp.

[11] Correa-Rodríguez M., Pocovi-Gerardino G., Callejas-Rubio J.-L., Fernández R.R., Martín-Amada M., Cruz-Caparros M.-G. (2019). The prognostic nutritional index and nutritional risk index are associated with disease activity in patients with systemic Lupus erythematosus. Nutrients.

[12] Mateos M.-V., Sonneveld P., Hungria V., Nooka A.K., Estell J.A., Barreto W. (2020). Daratumumab, Bortezomib, and Dexamethasone versus Bortezomib and Dexamethasone in patients with previously treated multiple myeloma: three-year follow-up of CASTOR. Clin Lymphoma Myeloma Leuk.

[13] Padala S.A., Barsouk A., Barsouk A., Rawla P., Vakiti A., Kolhe R. (2021). Epidemiology, staging, and management of Multiple myeloma. Med Sci (Basel).

[14] Derman B.A., Zha Y., Zimmerman T.M., Malloy R., Jakubowiak A., Bishop M.R. (2020). Regulatory T-cell depletion in the setting of autologous stem cell transplantation for multiple myeloma: pilot study. J Immunother Cancer.

[15] D'Souza A., Shah N., Rodriguez C., Voorhees P.M., Weisel K., Bueno O.F. (2022). A phase I first-in-Human study of ABBV-383, a B-cell maturation antigen × CD3 bispecific T-cell redirecting antibody, in patients with relapsed/refractory multiple myeloma. J Clin Oncol.

[16] Landgren O., Hultcrantz M., Diamond B., Lesokhin A.M., Mailankody S., Hassoun H. (2021). Safety and effectiveness of weekly Carfilzomib, Lenalidomide, Dexamethasone, and Daratumumab combination therapy for patients with newly diagnosed multiple myeloma: the Manhattan nonrandomized clinical trial. JAMA Oncol.

[17] Kuiper R., Zweegman S., Van Duin M., van Vliet M.H., van Beers E.H., Dumee B. (2020). Prognostic and predictive performance of R-ISS with SKY92 in older patients with multiple myeloma: the HOVON-87/NMSG-18 trial. Blood Adv.

[18] Perrot A., Facon T., Plesner T., Usmani S.Z., Kumar S., Bahlis N.J. (2021). Health-related quality of life in transplant-ineligible patients with newly diagnosed multiple myeloma: findings from the phase III MAIA trial. J Clin Oncol.

[19] Mateos M.V., Prosper F., Martin Sánchez J., Ocio E.M., Oriol A., Motlló C. (2023). Phase I study of plitidepsin in combination with bortezomib and dexamethasone in patients with relapsed/refractory multiple myeloma. Cancer Med.

[20] Perez C., Botta C., Zabaleta A., Puig N., Cedena M.-T., Goicoechea I. (2020). Immunogenomic identification and characterization of granulocytic myeloid-derived suppressor cells in multiple myeloma. Blood.

[21] Li Y., Wang W., Yang F., Xu Y., Feng C., Zhao Y. (2019). The regulatory roles of neutrophils in adaptive immunity. Cell Commun Signal.

[22] Xue A., Zhang H., Song S., Yu X. (2024). Effects of N-acetylcysteine combined with Ambroxol Hydrochloride on clinical symptoms, CRP, and PCT in children with pneumonia. Clinics (Sao Paulo).

[23] Jeon J., Sung S., Moon Y., Koo J., Hyun K., Han K. (2021). Comparison of early postoperative cytokine changes in patients undergoing intubated and non-intubated thoracic surgery: a randomized controlled trial. Interact Cardiovasc Thorac Surg.

